# Field DCP testing, MSEW analysis, and monitoring-based investigation of a reinforced earth retaining wall collapse

**DOI:** 10.1371/journal.pone.0332879

**Published:** 2025-09-22

**Authors:** Minwoo Kim

**Affiliations:** Department of Civil Engineering, Chung-Ang University, Seoul, Republic of Korea; University of Sharjah, UNITED ARAB EMIRATES

## Abstract

Reinforced retaining walls are critical components of urban infrastructure, yet failures continue to occur due to complex geotechnical and environmental factors. This study investigates a collapse case, focusing on the influence of water infiltration from a damaged sewage pipe near a manhole. Photo documentation and portable dynamic cone penetration (DCP) testing were conducted to evaluate subgrade strength and identify localized weaknesses. Results revealed significant variability in ground rigidity, with particularly low resistance values near the manhole, indicating deterioration caused by seepage and soil softening. Stability analysis using MSEW software, under both static and seismic conditions, confirmed that affected wall sections did not meet safety requirements. Based on these findings, targeted remediation measures are proposed, including soil nailing, low-pressure grouting, and embankment reinforcement, complemented by continuous monitoring using displacement targets and inclinometers. This integrated approach offers both diagnostic insight and practical strategies to improve the design, maintenance, and resilience of MSE retaining walls, providing valuable guidance for engineers and decision-makers in preventing similar failures in densely built urban environments.

## Introduction

Reinforced earth retaining walls, often referred to as mechanically stabilized earth walls, have firmly established themselves as cornerstone elements in the realms of construction and urban development. These structures combine soil and reinforcement to provide high stability. Their design allows them to bear and counteract significant external loads and pressures, often originating from the very terrains they are built within [1]. Their adoption has expanded due to proven resilience and adaptability. These walls can seamlessly blend with diverse terrains, be it rocky hillsides or sloped urban landscapes, making them a preferred choice for engineers and architects. They are particularly valuable in regions with varied topography and steep gradients [2].

The defining characteristic of these reinforced earth retaining walls is undeniably their sophisticated load distribution mechanism [3]. This unique capability ensures that they stand firm and stable even when subjected to uneven and substantial pressures. Over the years, the amalgamation of their distinctive design and mechanical prowess has enabled them to carve a niche in a myriad of infrastructural endeavors. They are used in highways, bridges, and residential developments. Here, engineers and architects have seamlessly combined the reinforced walls not just for structural soundness but also to complement the overall aesthetic appeal, a reflection of the harmonious blend of form and function [4–6].

Despite careful design, reinforced earth retaining walls can still succumb to structural shortcomings [7,8]. Instances of their unexpected collapses have been reported, posing not only significant economic burdens but also raising alarming safety red flags for nearby inhabitants and infrastructure [9–11]. These unfortunate events underline the indispensable nature of regular and comprehensive inspections. Furthermore, to mitigate such failures, there’s an exigent requirement to delve deeper into the myriad causes and mechanisms that can trigger these collapses. It’s equally crucial to craft and implement robust preventive strategies that can avert potential catastrophes in the future, safeguarding both lives and investments.

While prior studies on reinforced earth retaining walls have examined stability issues using field testing methods such as the Dynamic Cone Penetration (DCP) test and computational tools like mechanically stabilized earth wall (MSEW), they often focus on either laboratory-controlled conditions or single-site case studies without integrating multiple diagnostic approaches [12,13]. Our study distinguishes itself by combining multi-site DCP field data with detailed photographic documentation and MSEW-based limit equilibrium modeling to establish a direct correlation between subsurface rigidity variations, water infiltration pathways, and structural stability degradation. Unlike earlier works that primarily assess load-bearing capacity or reinforcement performance in isolation [12,14–16], this research emphasizes the role of localized infrastructural defects—such as ruptured utility lines and manhole seepage—in triggering instability. By integrating empirical measurements, visual inspections, and computational analysis, we present a holistic diagnostic framework that can be adapted for proactive monitoring and remedial design in urban retaining wall applications.

This study aims to systematically investigate the causes and contributing factors of a reinforced earth retaining wall collapse through an integrated methodology that combines field DCP testing, MSEW numerical analysis, literature review, and targeted in-situ monitoring recommendations. The research objectives are fourfold: (1) to evaluate variability in subgrade strength and detect localized weaknesses using high-resolution DCP testing; (2) to analyze structural stability under actual site conditions through MSEW modelling, accounting for geotechnical and hydrological influences; (3) to identify the role of drainage deficiencies and utility-related water infiltration in accelerating wall failure; and (4) to propose data-driven remedial measures and monitoring strategies, including hybrid reinforcement systems and continuous displacement and tiltmeter surveillance. To comprehensively address the complexities of reinforced earth retaining walls, the study first grounds its investigation in a critical review of relevant literature and technical documentation, ensuring alignment with established knowledge and prior findings. This is complemented by hands-on site evaluations that provide direct insights into field conditions and potential vulnerabilities [17,18]. The field program integrates portable dynamic penetration tests to deliver precise, localized assessments of soil strength and variability, which, in conjunction with MSEW simulations, yield a nuanced understanding of stability performance [19–21]. By merging theoretical insights, empirical field data, and numerical modelling, the study provides a comprehensive, evidence-based assessment of the collapse and offers actionable guidance for engineers and policymakers to improve the design, maintenance, and long-term resilience of similar urban retaining wall structures.

Despite their widespread use in urban construction, reinforced earth retaining walls remain insufficiently studied in both academic and practical contexts, particularly regarding failure mechanisms and mitigation strategies. This study addresses this gap through a multidisciplinary approach that integrates theoretical analysis, field inspections, and empirical testing. By examining these walls from design to real-world performance, the research aims to identify vulnerabilities, enhance structural integrity, and extend service life. The findings reaffirm their critical role in infrastructure while emphasizing the need for continual improvements in design, maintenance, and resilience against urbanization pressures.

## Literature review

### Concepts and principles of retaining wall

Unlike rigid construction materials such as rock, steel, or concrete, soil is a discontinuous medium with weak particle bonding, making it prone to failure under load. Henri Vidal’s experiments with dry sand and pine needles demonstrated that reinforcement can significantly improve soil stability by enabling higher stacking and greater resistance to external forces [22]. This concept—combining soil with materials of distinct mechanical properties—forms the foundation of reinforced earth retaining walls. Reinforcement layers, placed at regular intervals, mobilize frictional resistance and tensile strength, creating a composite mass that behaves as a single structural unit with superior load-bearing capacity.

### Design of retaining wall

The concept and principle of retaining walls were first introduced by Henri Vidal in France in 1963 [23]. Since then, numerous analytical and design methods have been suggested for the logical design of retaining walls [4,24–26]. Most employ limit equilibrium analysis to establish the safety factor for the wall’s failure using the Allowable Stress Design (ASD) approach [11,27–29]. There are two primary limit equilibrium analysis design methods [30]. The first is the tie-back analysis method, which equates the horizontal active force of the reinforced earth with the horizontal resistance force generated by the reinforcement [31]. The second method involves considering the reinforcement’s effect when analyzing force or moment equilibrium on an assumed failure plane, such as traditional slope stability analysis [32].

Prominent design methods for reinforced earth retaining walls are the FHWA method (Federal Highway Administration, 2017) and NCMA method (National Concrete Masonry Association, 1998) in the US, the BS method [33] in the UK, and the Japan Civil Engineering Research Center method [34]. In Korea, the design method for reinforced retaining walls using the allowable stress design, influenced by the U.S. FHWA method, is prevalent. This method is straightforward and ensures safety.

In synthesizing these design approaches, it becomes evident that while international methods—such as FHWA, NCMA, BS, and JCERC—share a common reliance on limit equilibrium principles, they differ in safety factors, reinforcement considerations, and adaptability to local soil conditions. The Korean adoption of the FHWA-based allowable stress design reflects a preference for simplicity and proven safety, yet may not fully address site-specific challenges such as varying seismic demands or non-standard backfill materials. This study builds on these insights by evaluating existing design frameworks in light of observed field failures, aiming to bridge the gap between theoretical safety assumptions and real-world performance.

### Destruction of retaining wall

Reinforced retaining walls are critical in counteracting gravitational and lateral earth pressures in applications such as roadways and hillside developments. However, their stability can be compromised by hydrostatic pressure, poor drainage, inadequate reinforcement design, substandard materials, or construction defects [9,10,35–38]. Proper drainage systems and strict adherence to design standards are essential to prevent such failures.

Design-related issues include improper reinforcement length. Kong, Oh [10] investigated the failure of these structures based on reinforcement length. Their study pointed out that while reinforcements enhance both safety and economic efficiency, using them excessively can be counterproductive economically. Their analysis emphasized the different behaviors of straight and curved sections when different reinforcement lengths are used, recommending that straight and curved sections require unique optimization of reinforcement lengths to achieve the best performance. An innovative approach towards sustainability in reinforced soil walls is presented by Vibha and Divya [35]. They emphasized the potential of construction and demolition waste (CDW) as backfill for geosynthetic-reinforced soil walls due to the scarcity of ideal granular materials. CDW’s geotechnical properties met the required specifications for ideal backfill material for mechanically stabilized earth walls, shedding light on sustainable solutions in retaining wall construction.

Xu, Chen [36] utilized a novel visual model to explore the failure mechanisms of cohesionless narrow backfills installed behind rigid retaining walls. Their research provided insights into the effects of backfill width and retaining wall inclination on failure mechanisms. Notably, they observed that traditional calculations often underestimate the active earth pressure, indicating that more comprehensive methodologies might be necessary for accurate predictions. Do, Kim [37] provide a historical perspective on reinforced earth retaining walls used in expressways. Their research pinpoints minor defects across the design, construction, and maintenance phases as primary reasons for the damage observed in such walls. They emphasize the necessity of evolving perceptions regarding the importance of each stage in the construction process. Binici, Temiz [9] discussed a case study where a retaining wall collapsed, highlighting the critical importance of material quality and workmanship. This research serves as a stark reminder of the repercussions of poor construction practices and design. Specifically, the failure in this case was attributed to non-compliance with the TS 7984 Turkish standard, stressing the value of adhering to industry standards. Lastly, [38] conducted shaking table tests on geogrid reinforced soil retaining walls to analyze their behavior under seismic loading. They proposed a lateral displacement control index based on observed failure modes, further defining damage-assessment criteria, which can be instrumental in regions prone to seismic activities.

In synthesizing these studies, it is clear that retaining wall failures stem from an interplay of design, material, environmental, and construction factors. Research on reinforcement length optimization highlights the importance of tailoring design to wall geometry, while sustainable backfill alternatives such as CDW offer promising yet underexplored solutions. Findings on backfill geometry and wall inclination challenge conventional earth pressure assumptions, suggesting a need for refined analytical models. Historical case analyses and seismic performance studies further emphasize that quality control, adherence to standards, and context-specific design are critical for long-term stability. By integrating these perspectives, this study aims to connect failure mechanisms with practical mitigation strategies, advancing both the technical robustness and sustainability of reinforced retaining wall design. In conclusion, reinforced retaining walls, while effective and economically efficient, require meticulous attention to factors ranging from reinforcement length to material quality and adherence to design standards. Recent studies also underscore the importance of sustainable solutions and accurate assessments of the structures’ behavior under different stressors, including seismic activities.

## Methodology

This study is part of the “Investigation of Failure Causes and Countermeasures for Reinforced Earth Retaining Walls in Apartment Complexes,” conducted by the Korean Geosynthetics Society (KGSS) for submission to Jungheung Construction Co., Ltd. The author participated in the research and obtained academic research permission from the relevant stakeholders. As the field site was privately owned by Jungheung Construction and the investigation was carried out at their request, no additional governmental or public permits were required.

The inquiry into the collapsed retaining wall was steered using a systematic approach that integrated both qualitative data analysis and on-ground examinations. Initially, a comprehensive review of the available data was conducted. The focal point here was understanding the wall’s intended design and its actual construction. This step entailed the close examination of design blueprints, geotechnical survey reports, construction diaries, and the real-time construction scenario of the neighboring structures. Subsequent to the data evaluation, a physical inspection of the collapsed section of the retaining wall was carried out. This procedure aimed to ascertain the current condition of the site, particularly any deviations caused by extraneous environmental elements. It has been identified that such external influences can significantly impact the integrity of civil structures [39]. Lastly, to ensure an all-encompassing understanding, a portable dynamic cone penetration test was utilized. This instrument, as documented by previous works [40,41], is adept at rapidly measuring the relative density or strength of the involved ground, thereby presenting a clearer picture of the extent of ground disruption.

The rationale behind the chosen methodology was to ensure a holistic understanding of the problem, enabling the drawing of actionable insights. The structured approach amalgamates desk research and fieldwork, providing both depth and breadth to the study. Reviewing extant data serves as the foundation, as it gives a clear blueprint of the intended design and the initial construction parameters. Analyzing these documents can identify discrepancies between design intentions and real-world implementation. Direct on-site inspections act as a reality check [17,18]. Physically examining the site can reveal tangible issues and environmental factors that might not be evident in design blueprints or construction records. The dynamic cone penetration test was chosen because of its precision in evaluating ground conditions, which is imperative in such collapse investigations [19–21]. The instrument’s ability to swiftly assess the ground’s density and strength can spotlight regions that may be compromised, thus identifying potential weak spots [42]. Moreover, The portable DCP Test was selected over alternative geotechnical characterization methods such as the Standard Penetration Test (SPT) and Cone Penetration Test (CPT) due to its proven efficiency, cost-effectiveness, and adaptability to the constrained urban environments where the retaining wall failures occurred. Unlike SPT, which requires bulky drilling equipment and is time-consuming, or CPT, which demands specialized rigs and is less suitable for gravelly or mixed-fill soils, the portable DCP can be deployed rapidly with minimal disturbance and without heavy machinery, making it ideal for post-failure diagnostic work in areas with limited accessibility [43–45]. Furthermore, the DCP has been shown to provide reliable correlations with soil strength parameters, California Bearing Ratio (CBR), and relative density, enabling effective evaluation of subsurface rigidity and moisture-induced weakening in situ [46,47]. These advantages, combined with the need for immediate and repeated measurements across multiple urban sites, made the DCP the most practical and scientifically appropriate choice for this investigation.

### Document review and foundational assessment

The retaining wall’s design and construction materials were procured and subjected to a rigorous evaluation. On the surface, the design and construction appeared well-founded with indications of thorough structural stability checks, potential reinforcement of foundations, and diligent compaction processes near the structure. The strategic placement of drainage ditches further added to its prima facie stability. However, detailed scrutiny revealed some concerning inconsistencies.

One notable discovery was the damage to the sewage pipe connecting Building 109 to the main sewage manhole. The resultant sewage leakage, combined with the infiltrative channels that developed thereafter, was seen as a significant factor that might have precipitated the collapse of the retaining wall.

To substantiate the structural stability of the retaining wall, the renowned ‘MSEW’ software was employed. Designed especially for the analysis of reinforced earth retaining walls, this tool is a staple in many design processes involved in erecting such walls [48–51].

For the MSEW (limit-equilibrium) analyses we defined soil parameters from site data and DCP-derived correlations (Nd → SPT N; N → φ, c). In the collapse-affected reinforced fill adjacent to the manhole, where average Nd ≈ 2–6 m depth, we adopted a conservative friction angle φ = 20° and cohesion c = 0 kPa (granular, strength loss due to infiltration). For intact reinforced/backfill zones (Nd > 10), we used φ = 32–34°, c = 0 kPa (well-compacted granular). For the foundation soil beneath the facing, we used φ = 28°, c = 10–15 kPa to reflect slightly plastic subgrade inferred from records and field observations. Interface friction was δ = 0.67φ (default MSEW practice), and base sliding resistance used μ = tan δ. Reinforcement pullout/rupture capacities followed manufacturer properties and MSEW reduction factors. We applied a uniform surcharge of 20 kPa at the crest (dead load of surface fill/pavement ≈ 8–10 kPa plus live load 10–12 kPa for light vehicular access), extending 3 m behind the facing. Boundary conditions: horizontal backfill behind the reinforced zone; vertical modular facing; foundation assumed level, with bearing checked at the toe; active/at-rest coefficients computed internally by MSEW from the specified φ. Groundwater assumptions: baseline checks assumed dry (no positive pore pressure) consistent with routine operation; event-based sensitivity cases imposed a perched phreatic surface at mid-height of the reinforced zone and ru = 0.2 within the collapse sector to simulate seepage from the ruptured sewer.

A comprehensive evaluation using this software across various sections and heights of the wall yielded largely positive results. The wall demonstrated resilience against potential hazards such as activity, overturning, bearing capacity, pullout, and fracture.

Table 1 presents the results of a structural review for a reinforced earth retaining wall with a height of H = 14.0m, based on existing data. The table outlines the safety rate or safety factor (Fs) values under various activities or conditions the wall might be subjected to, such as conduction, support, drawing, and fracture. Each value is compared to a benchmark safety rate to determine if the structure’s condition under each activity is within a safe range.

**Table 1:** Structural Safety Factors for a Reinforced Earth Retaining Wall.

Assessment	Safety Rate (Fs)				
	Activities	Conduction	Support	Drawing	Fracture
H = 14.0m	2.304 > 1.5	4.20 > 2.0	4.78 ≥ 2.5	2.919 > 1.5	2.023 > 1.5

**Fig 1:**
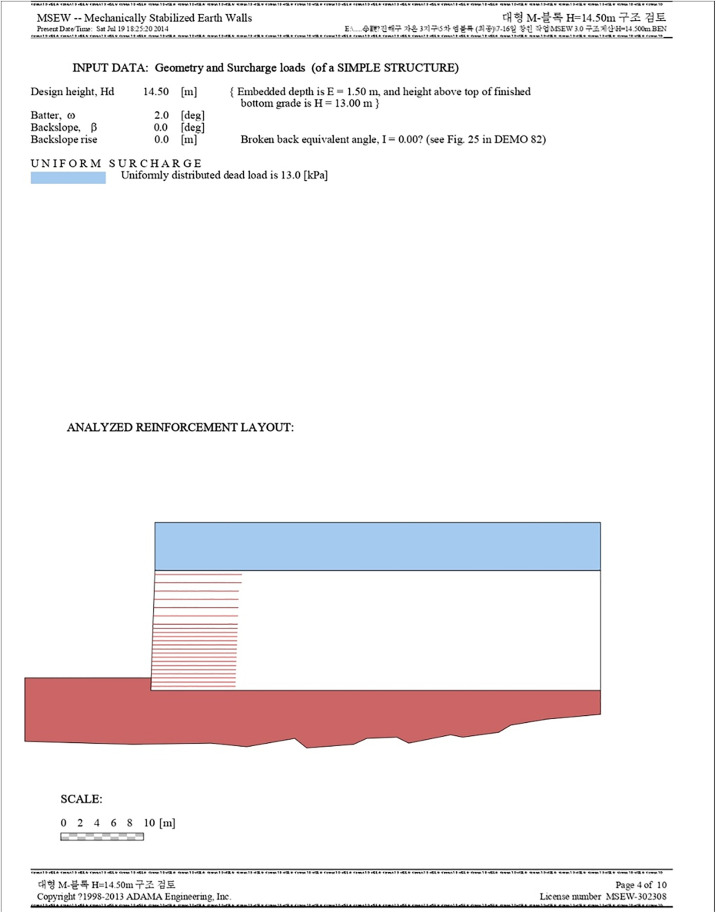
Structural Review Result of Reinforced Retaining Wall (Reinforcement Layout). The light blue zone represents the uniformly distributed surcharge load area with a dead load of 13.0 kPa applied above the retained fill; the white zone indicates the retained backfill composed of engineered fill reinforced with horizontal geogrid layers; the horizontal red lines depict the individual geogrid reinforcement layers placed at specified spacing to ensure slope stability; the dark red zone illustrates the foundation soil or original ground beneath the reinforced structure, while the irregular red base outlines the existing undulating ground profile prior to construction; black outlines mark the boundaries between different soil zones and structural components; and the scale bar at the bottom provides horizontal distance in meters for proportion reference.

Fig 1 represents an output from the MSEW software, showcasing the design and analysis of a sample structure. The provided input data delineates parameters like the design height, base width, back slope, and surcharge values, among others. The visual part of the diagram highlights a cross-sectional view of the wall, where two distinct layers can be observed. The top layer, indicated in blue, represents a uniformly distributed surcharge, while the bottom section, depicted in red with horizontally striped patterns, shows the analyzed reinforcement layout of the wall. This layout is structured to ensure the wall’s mechanical stability. A scale provided at the bottom gives a perspective on the actual dimensions of the wall, spanning from 0 to 10 meters.

**Fig 2:**
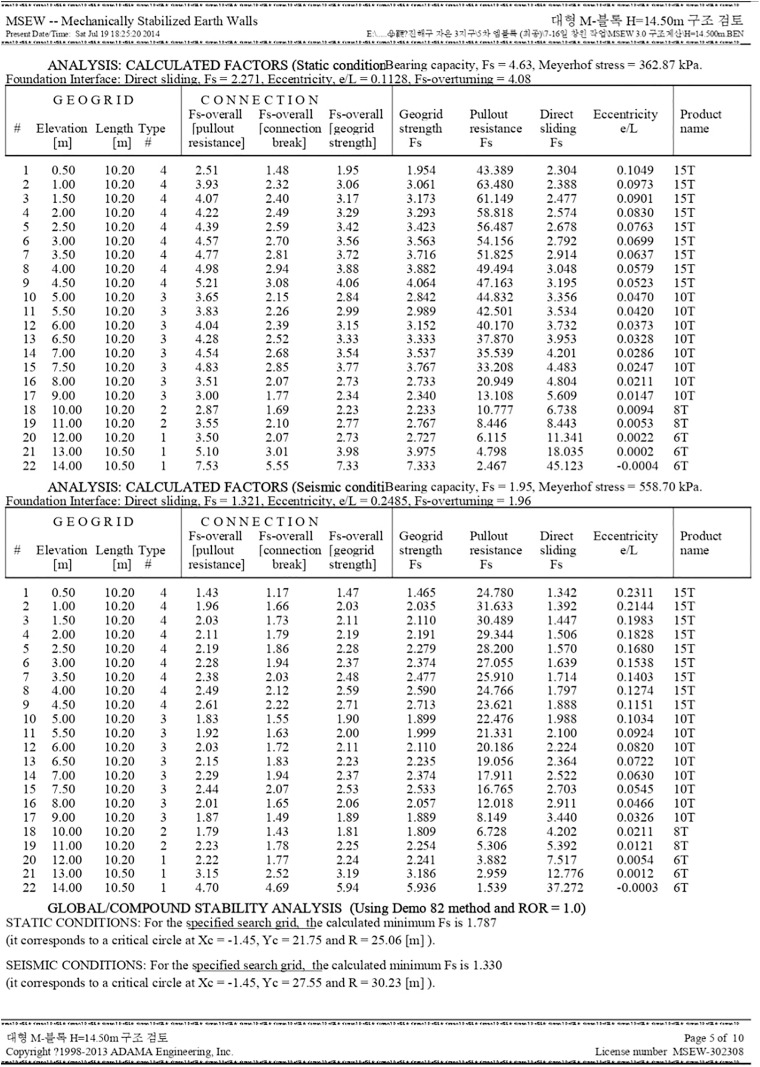
Structural Review Result of Reinforced Retaining Wall (Calculated Factors).

Fig 2 provides a comprehensive analysis of calculated factors under different conditions: static and seismic. This analysis pertains to the structural integrity and safety measures of the studied geogrid. The table is segmented into multiple sections for clarity: The first section showcases calculated factors under static conditions. Factors like elevation, length type, tensile strength, forcewall pullout, forcewall geometrical strength, ground pullout, direct sliding, and eccentricity are tabulated against their corresponding values. The product type, labeled as ‘1ST’, is consistently noted for each row. The subsequent section is a continuation of the table and represents data under seismic conditions. This section, similar to the static condition analysis, lays out the calculated factors, but now considering the potential disturbances introduced by seismic activities. At the bottom, a brief note indicates the method used for global compound stability analysis, citing the Demo8 method. There’s also a mention of the static conditions for the specified parameters and the resultant safety factor of 1.348.

The MSEW analysis results reveal that the reinforced soil wall at the collapse location exhibited a factor of safety below the acceptable threshold, indicating insufficient stability under the given loading and drainage conditions. The reduction in stability was most pronounced in sections where reinforcement length and tensile capacity were marginal relative to the active wedge geometry, and where pore water pressures were likely elevated due to inadequate drainage. In contrast, MSEW simulations for sections farther from the failure zone yielded factors of safety within acceptable design limits, reflecting adequate reinforcement distribution and shear resistance. The modeled critical slip surfaces in the failure zone aligned closely with the observed collapse profile, confirming that localized strength loss within the reinforced zone—likely aggravated by water infiltration—was the primary driver of failure. These results highlight the need for improved drainage, optimized reinforcement layout, and consideration of potential water-induced softening in future wall designs.

**Fig 3:**
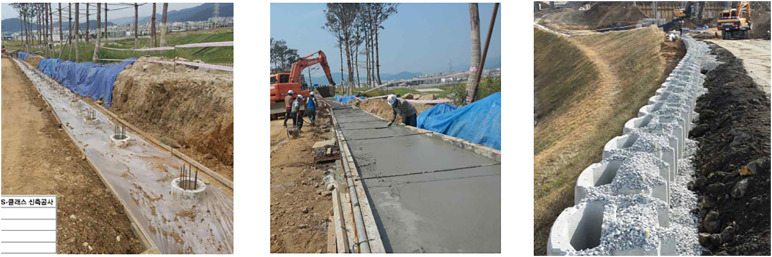
Construction Progress by Process.

The investigation delved deeper into the construction data analysis. This involved examining test reports on materials used during the wall’s construction, evaluating post-construction quality tests, and reviewing photographs taken during various construction stages. Both the material quality tests and the post-construction quality tests at the collapse site met the standards expected for a reinforced earth retaining wall. Analysis of the construction phase confirmed that a pile foundation had been integrated beneath the foundational concrete to ensure solid support. Moreover, thorough compaction was carried out using specialized equipment. Through photographic analysis, the construction progress of the reinforced earth retaining wall was verified, reiterating that a pile foundation was present beneath the foundational concrete, and comprehensive compaction was achieved with the appropriate equipment. Fig 3 illustrates the construction progress by process.

### On-site inspection

#### Survey.

Following the unexpected collapse, a structured on-site inspection was conducted to identify the causes of failure and inform emergency stabilization measures. Visual assessment of the upper section of the reinforced earth retaining wall revealed a ruptured sewage pipeline. The continuous leakage likely led to localized soil saturation, reduced shear strength, and progressive loss of internal stability in the reinforced zone. The damaged pipe was promptly repaired to restore proper drainage and prevent further seepage.

Closer inspection of the wall face indicated several modular blocks that had become destabilized due to loss of backfill support. These blocks were at high risk of detachment, posing immediate safety hazards. As a temporary measure, unstable blocks were carefully removed or repositioned, and compacted fill was placed to restore minimal front-face integrity.

Given the high permeability of the collapsed section and the forecast of rainfall, impermeable tarpaulin sheets were installed over the crest and the exposed wall face to minimize additional infiltration. This measure aimed to limit pore water pressure buildup, which could otherwise trigger secondary failures.

**Fig 4:**
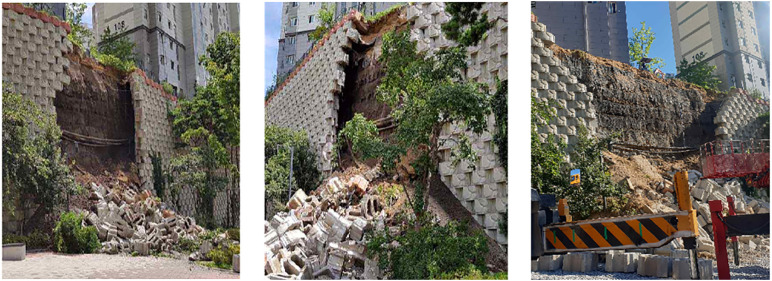
Collapse status of reinforced retaining walls.

**Fig 5:**
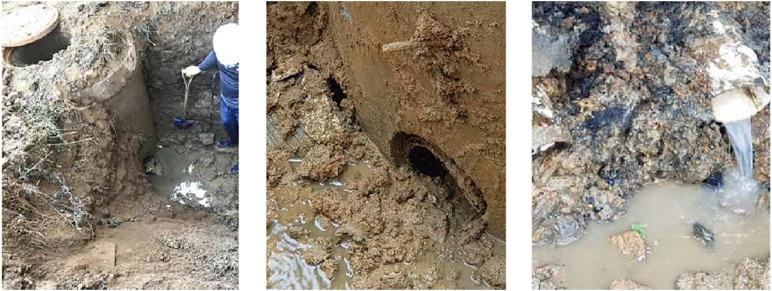
Sewer line breaks and repairs.

Figs 4 and 5 illustrate the observed structural damage, the failed sewer system, and emergency mitigation actions. The combined evidence suggests that inadequate drainage maintenance, compounded by prolonged seepage, was a primary contributor to the collapse mechanism.

### Dynamic cone penetration test

#### DCP test procedure.

The portable DCP test was employed to assess subsurface strength and identify variations in soil compaction near the collapse zone. This portable device allows rapid in-situ evaluation of soil resistance with minimal equipment, making it suitable for constrained or unstable areas (see Appendix A1 for details).

**Fig 6:**
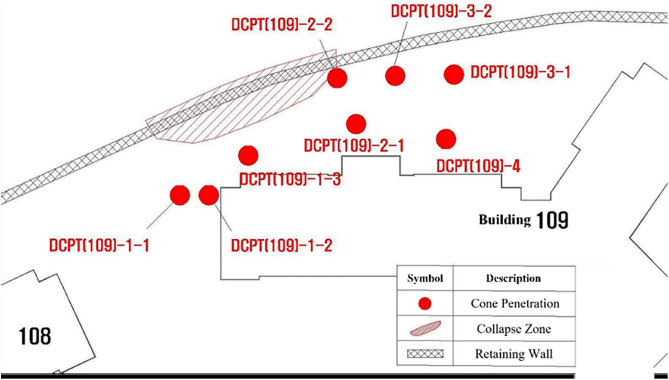
Penetration Point.

Eight test locations were selected to capture soil strength profiles across different structural contexts and to ensure representative coverage of the affected area. The spacing between points ranged from 1.5 m to 3.0 m, reflecting changes in wall geometry and proximity to visible distress zones. Two points adjacent to the collapse and manhole (DCPT(109)-1, 2) were chosen to assess soil conditions where maximum deformation was observed. One point within the reinforced zone near the collapse (DCPT(109)-3) was located approximately 2 m from the failure plane to evaluate reinforcement influence on soil strength. A further point outside the reinforced zone (DCPT(109)-4), positioned 5 m from the collapse, served as a control. The distribution and spacing were determined based on the wall’s total length (~15 m) and the need to achieve adequate spatial resolution for comparative analysis of localized versus unaffected areas. The arrangement of test points and their spatial relation to the retaining wall are illustrated in Fig 6.

#### DCP test results.

The Portable DCP test results provide a comprehensive understanding of the subsurface conditions across various locations. The summarized results are presented as follows:

DCPT(109)-1–1, 2: Positioned to the left of apartment 109, the results for DCPT(109)-1–1 and 1–2 showed an Nd value exceeding 10 in the 0.5-2.5m range from the surface. This suggests the top layer was loosely to moderately compacted. Beyond the depth of 2.0m, the compacted layer was so dense that the probe could not penetrate further.

DCPT(109)-1–3: This test was performed near the manhole (MC-10), the site of the DCPT sewer line rupture. With Nd values under 10, extending down to about 6 meters, the results indicated a ground of loose to very loose relative density. Such results hint at significant strength degradation, possibly due to wastewater leakage from the broken sewer line seeping to the lower sections of the manhole. The consequent reduction in the overall strength of the ground demands remedial action to restore ground strength between the manhole and the apartment wall.

DCPT(109)-2−1, 2: Tests for DCPT(109)-2−1 and 2−2 were conducted next to the right side of the collapsed area and near the reinforced earth retaining wall, respectively. While the landscaped region above the reinforcement presented a loose relative density with Nd values under 10, depths below 2 meters from the reinforcement registered a dense relative density with Nd values exceeding 10.

DCPT(109)-3−1, 2: This assessment aimed to discern any strength variations with increasing distance from the collapse zone. The findings paralleled those of DCPT(109)-2, suggesting no discernible ground deformation apart from the collapsed area.

DCPT(109)-4: DCP tests conducted on the embankment’s rear – an area beyond the reinforcement’s influence – revealed that both the reinforcement and the backing embankment were thoroughly compacted, displaying no deformation signs.

The interpretation of Nd values from the portable DCP test followed established correlations between penetration resistance and relative soil density [46,47]. For granular soils, Nd values less than 5 blows/10 cm were classified as loose, 5–10 as moderately dense, and greater than 10 as dense. For cohesive soils, lower Nd values were indicative of reduced undrained shear strength, with <5 suggesting very soft to soft conditions. In the study sites, localized zones recorded Nd values below 5, particularly adjacent to areas of observed water infiltration from ruptured utilities and manhole seepage. These low-resistance zones were identified as primary contributors to loss of bearing capacity and progressive deformation. Consequently, targeted remedial measures were proposed: grouting for void filling and strength restoration in soft, water-compromised zones, and soil nailing for improving stability where moderate resistance (Nd 5–10) was recorded along critical slip surfaces. Synthesizing the portable DCP test findings, in the region of the collapse, the reinforcing soil manifests a very loose to loose relative density. Such conditions necessitate reinforcement interventions, likely in the form of grouting. Conversely, the reinforcing soil outside the collapse vicinity showcases a dense to very dense relative density. This density translates to adequate strength, obviating the need for separate reinforcement measures.

**Fig 7:**
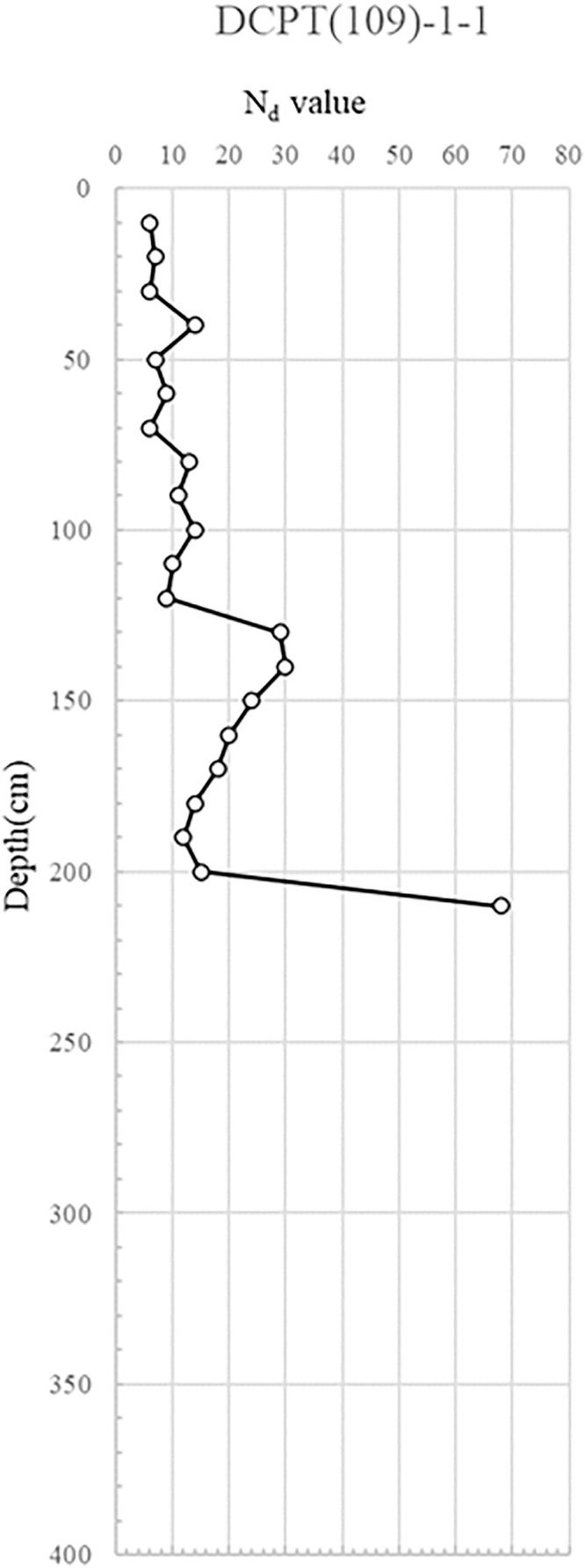
Portable Dynamic Cone Penetration Test Results (DCPT 109-1-1).

**Fig 8:**
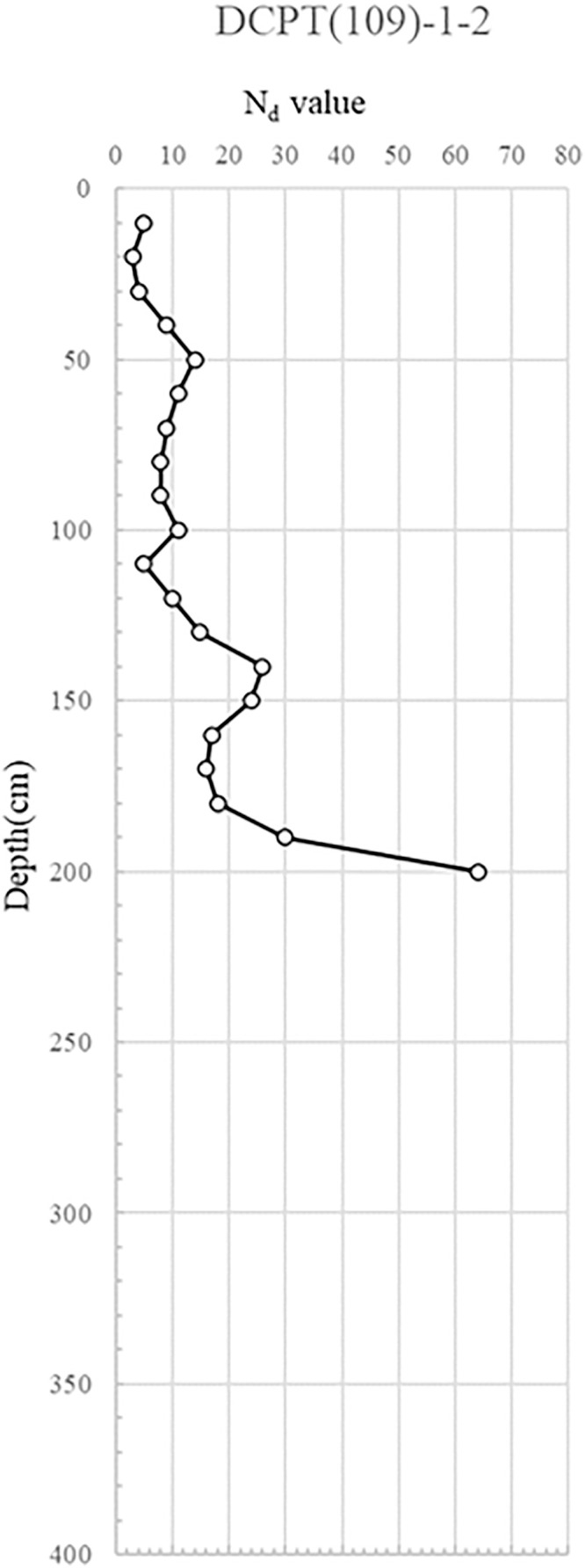
Portable Dynamic Cone Penetration Test Results (DCPT 109-1-2).

**Fig 9:**
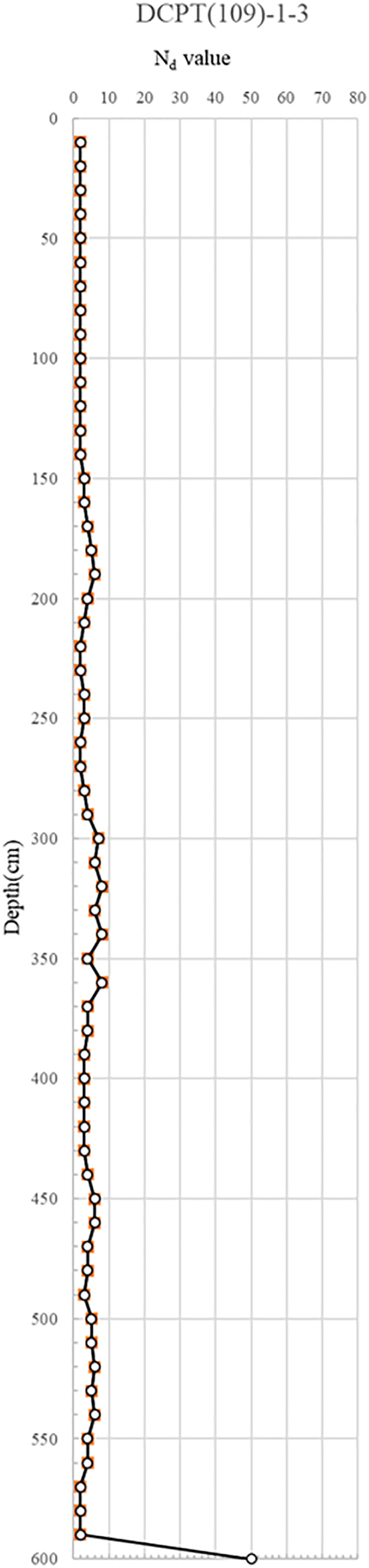
Portable Dynamic Cone Penetration Test Results (DCPT 109-1-3).

**Fig 10:**
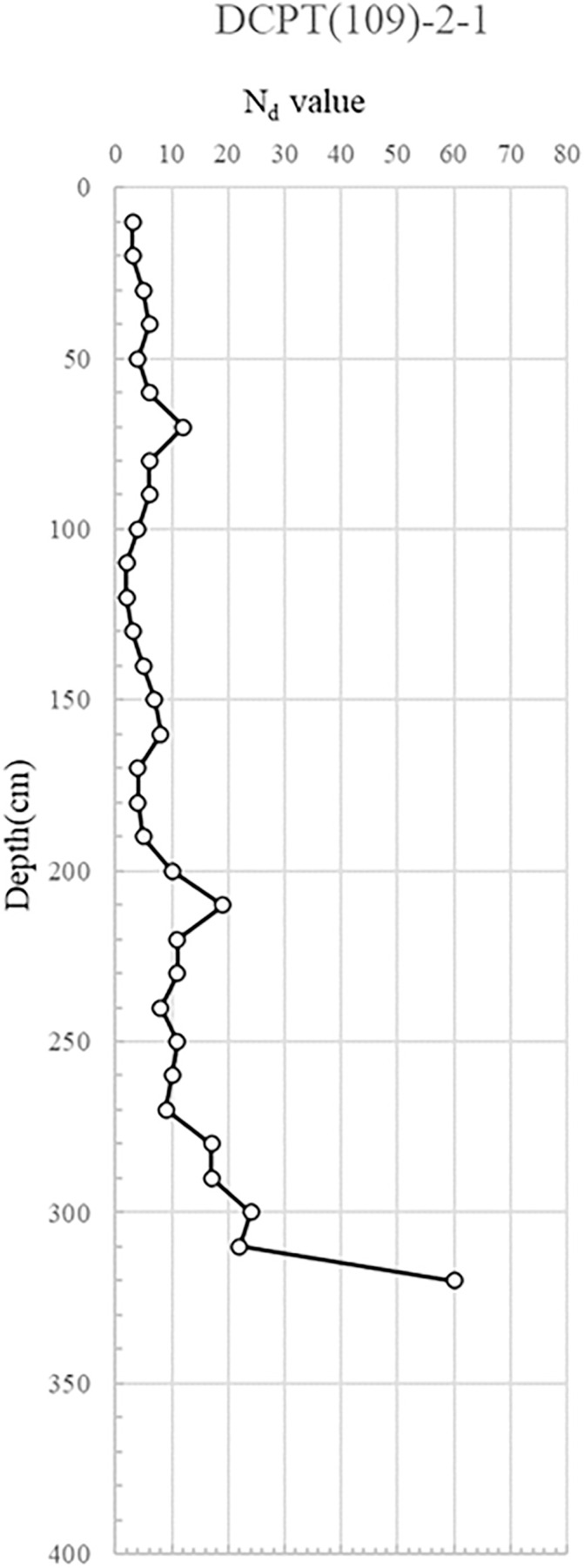
Portable Dynamic Cone Penetration Test Results (DCPT 109-2-1).

**Fig 11:**
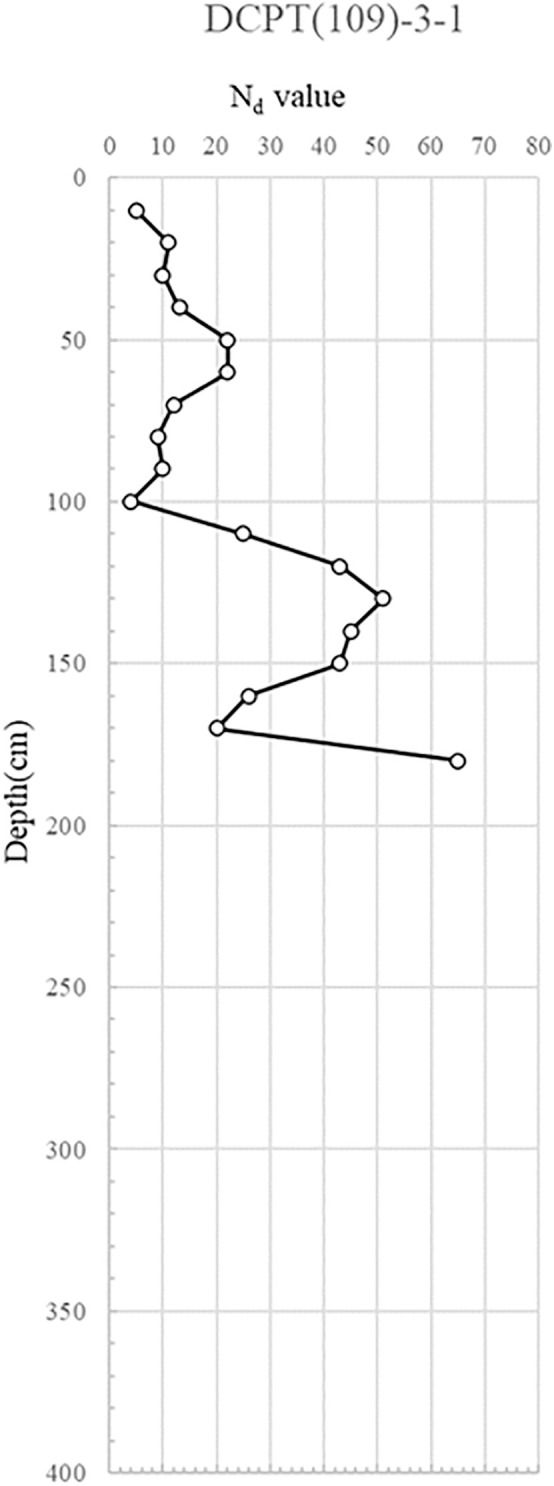
Portable Dynamic Cone Penetration Test Results (DCPT 109-3-1).

**Fig 12:**
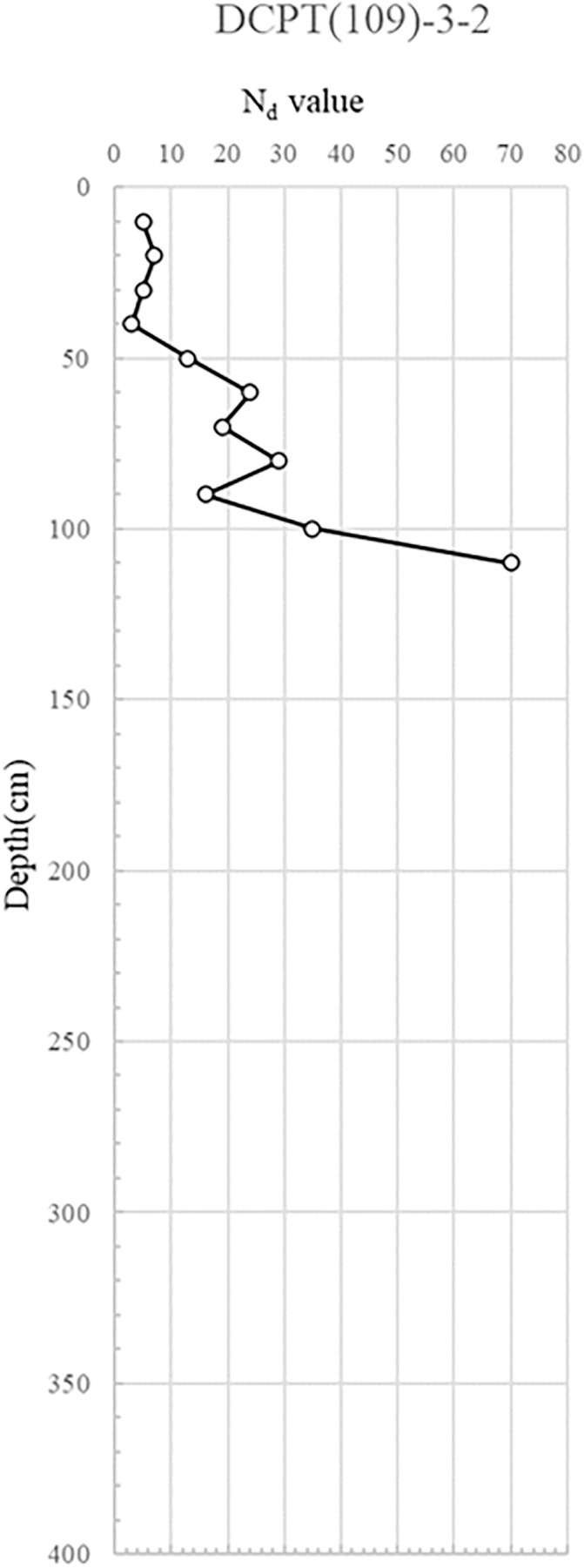
Portable Dynamic Cone Penetration Test Results (DCPT 109-3-2).

**Fig 13:**
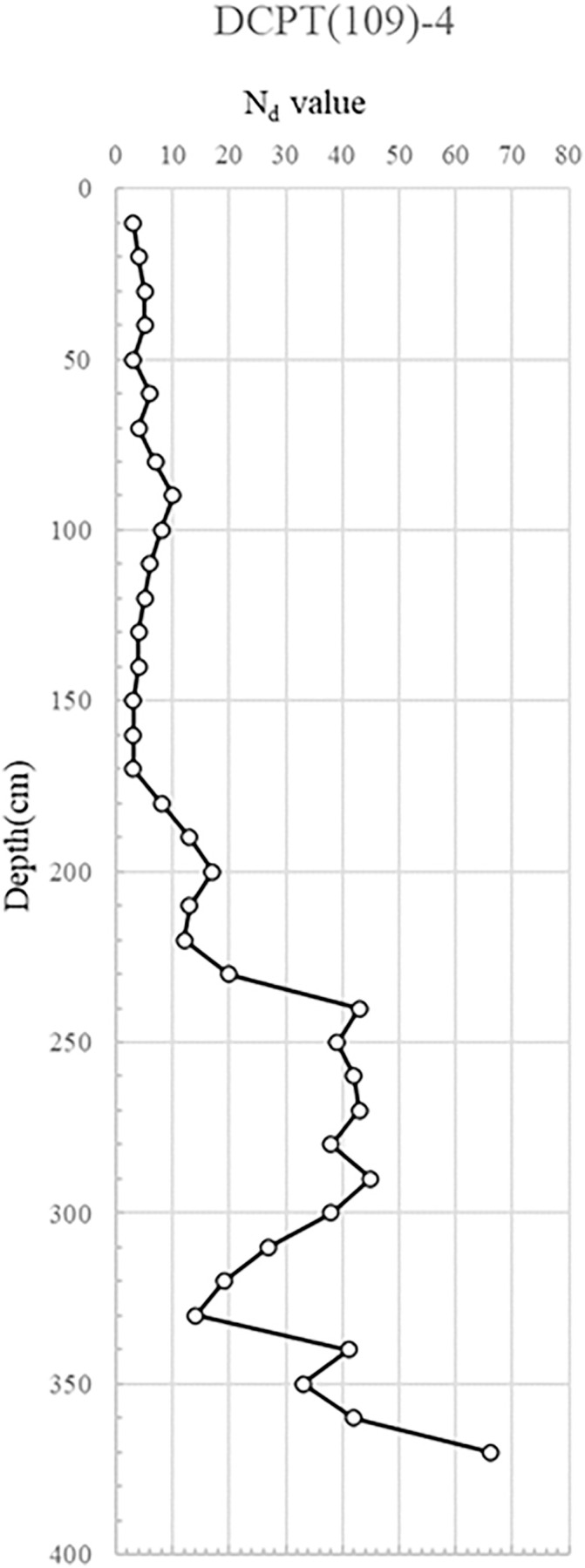
Portable Dynamic Cone Penetration Test Results (DCPT 109−4).

The DCP test results indicate distinct spatial variability in subsurface strength, with critically low Nd values (<5) concentrated near the manhole and ruptured sewer line, suggesting localized softening from prolonged water infiltration. This loss of relative density, extending to depths of 6 m, implies reduced bearing capacity and heightened susceptibility to settlement and lateral deformation. In contrast, areas outside the collapse zone exhibited dense to very dense conditions (Nd > 10), reflecting adequate compaction and structural integrity of the reinforced backfill. The juxtaposition of loose, water-compromised soils adjacent to structurally sound zones suggests a differential deformation mechanism, where localized strength loss triggered stress redistribution, progressive displacement, and eventual wall instability. The detailed test results are shown in Fig 7 and through 13, Figs 8–13.

### Suggestion

#### Measure for the stability of reinforced retaining walls.

For mitigating the issues surrounding wall collapses in reinforced retaining walls, it’s pivotal to adopt robust and effective rehabilitation measures. One of the most prevalent methods utilized is the combination of soil nailing, soil, and a frontal block. In instances where soil nailing might not offer enough stability, especially in the rear sections, ground reinforcement through grouting (low-pressure injection) is the chosen path. To ensure the steadfastness of these walls, the optimal route is often a blend of both traditional methods and innovative solutions. Soil nailing, for instance, not only offers reinforcement but also grants flexibility for adapting to the unique conditions of each site. Furthermore, when coupled with low-pressure grouting, it can provide the necessary reinforcement to areas that might be deficient in stability. The methods mentioned above, while effective, come with their own sets of challenges and requirements. For instance, while the block facades method demands the establishment of an anchor and a facade, the concrete facade method necessitates the removal and rebuilding of compromised retaining walls. However, irrespective of the method chosen, the primary objective remains consistent: to ensure that the reinforced retaining walls are stable, durable, and resilient against potential adversities.

Table 2 presents a comparative review of three primary ground stabilization methods for rehabilitating reinforced retaining walls—Soil Nailing & Block Facades, Anchor & Concrete Facade, and Removing and Rebuilding Damaged Retaining Walls—with method selection guided by geotechnical site conditions identified through DCP (Dynamic Cone Penetrometer) testing. For soils with low Nd values (<4 blows/100 mm) indicating loose or weakly compacted backfill, the soil nailing method is preferred due to its low relative cost, short construction period (2–4 weeks), and ability to improve subgrade strength through deformable reinforcement bars, low-pressure grouting, and flexible adaptation to variable field conditions. For moderate-density soils (Nd ≈ 4–8) with localized instability and an available competent anchor support layer, the anchor and concrete facade method offers a suitable option, providing medium-cost, medium-duration (3–5 weeks) reinforcement by mobilizing tensile resistance via prestressed anchors and concrete facades without requiring full wall replacement. In cases of severe Nd variability across depth, extensive structural deterioration, or non-uniform soil profiles, complete removal and rebuilding of the retaining wall is warranted, despite its higher cost, longest duration (6–10 weeks), and greater earthwork requirements. This quantitative decision framework ensures that the chosen rehabilitation method directly addresses the measured ground conditions, optimizing stability, durability, and cost-effectiveness.

**Table 2:** Comparison and Review of Rehabilitation Methods for Reinforced Retaining Walls.

Ground Stabilization Techniques	Soil nailing & Block Facades	Anchor & Concrete Facade	Removing and Rebuilding Damaged Retaining Walls
Overview	Increase the strength of the subgrade by inserting reinforcement (deformable rebar) into the subgrade.	Prestress the anchor to increase shear resistance due to tensile forces.	Demolish the damaged retaining wall and all of its reinforcing earth and reconstruct the retaining wall.
Construction Order	1. Install Thumbnailing 2. Construct the front walland reinforced earth retaining wall	1. Install anchors 2. Install the straps (supports) 3. Attach anchors after constructing the front wall	1. Install false facilities 2. Remove the defensive wall 3. Reconstruct reinforced earth retaining walls
Features	- Reinforce areas with damage - Reinforce the ground with soil nailing - Flexibly adapt to field conditions - Improve ground strength with grout	- Must be close to the support layer of the anchor body - Front wall (concrete) requires installation - Separate bandit required	- Requires facilities - Involves extensive earthwork - Adjacent structures may be difficult to construct - Longer recovery time - Complex recovery process
Relative Cost	Low	Medium	High
Relative Effectiveness	High for loose soils	High for moderate-density soils	Very high if wall is severely damaged
Duration	2–4 weeks	3–5 weeks	6–10 weeks
Remarks	Best for loose soils (Nd < 4), adaptable to varying field conditions	Requires competent anchor support layer; good for Nd ≈ 4–8	Best for severe Nd variability or structural failure; longest duration

Directly reinforce with earth for durability (no tensioning necessary) and choose an aesthetically pleasing soil nailing + block front wall.

Combine soil nailing on the back of the reinforcing earthwork with low-pressure grouting when reinforcement is insufficient.

### Measurement plan for assessing wall stability

To ensure the comprehensive evaluation of wall stability, a rigorous measurement plan has been designed, blending direct stability measures with advanced instrumentation. Such instruments serve to provide quantitative data on the deformation or behavior of structural grounds, aiding in alleviating potential concerns about structural stability. For this particular site, a comprehensive setup was envisioned, placing instruments across the entirety of the reinforced earth retaining wall, within the apartment structures, and particularly in areas exhibiting wall deformation. The subsequent management standard and measurement frequency were then determined based on these installations.

Table 3 outlines the measurement items and their associated purposes, particularly focusing on monitoring potential front wall collapses and deformations. Selection of measurement items is based on standard geotechnical and structural monitoring practices outlined in retaining wall stability assessment guidelines, prioritizing instruments that directly detect displacement and tilt, as these parameters are most indicative of potential failure modes in reinforced retaining walls and adjacent structures. Two primary measures are highlighted: the Displacement Target and Structures Inclinometer. The Displacement Target aims to keep track of shifts in the reinforced retaining walls during various construction stages. Its primary goal is to understand the wall’s behavior during the construction process, which in turn helps to guarantee its stability as construction advances. In the event of a front wall collapse or deformation, this measure helps evaluate the wall’s stability by determining the range and degree of these displacements. Moreover, insights into the wall’s behavior during the construction phase are instrumental in ensuring continual stability. On the other hand, the Structures Inclinometer focuses on detecting tilt-induced displacements in apartment walls. Its significance lies in measuring these displacements to both validate and ensure the structural stability of the apartments.

**Table 3:** Measurement Items and Measurement Content.

Measure	Measurements’ Purpose	Front Wall Collapse/Deformation
Displacement Target	- Monitor displacements in reinforced retaining walls across different construction phases. - Understand the behavior of the wall during construction, ensuring stability as the project proceeds.	- Evaluate the wall’s stability by determining the extent of collapses and displacements. - Understand construction-phase wall behaviors to enhance ongoing stability.
Structures Inclinometer	- Ascertain tilt-based displacements in apartment walls.	- Measure apartment wall displacements to validate and maintain structural stability.

Table 4 provides a detailed breakdown of the measurement frequency for two specific metrics: the Displacement Target and the Structures Inclinometer. Frequency settings follow empirical data from similar urban retaining wall rehabilitation projects, where displacement rates are highest during active construction and early post-construction. Daily measurements during construction and weekly checks immediately after completion align with risk-based monitoring protocols in national geotechnical standards. For both measures, during the preconstruction phase, assessments are conducted once every two weeks. During the active construction phase, daily measurements are taken to closely monitor any potential changes or shifts. Once construction is completed, measurements are more frequent immediately post-construction, being taken weekly. However, as time progresses and the construction settles, this frequency decreases to monthly checks. An important note accompanying the table highlights the adaptability of this measurement schedule. The frequency can be adjusted based on the observed displacement trends and the unique conditions of each construction site. This flexibility ensures that monitoring is responsive and tailored to the specific needs and observations at each site.

**Table 4:** Measurement Frequency.

Measure	Preconstruction	Under Construction	Post Construction	Notes
Displacement Target	Once every 2 weeks	Once daily	Immediately post-construction: Weekly Subsequently: Monthly	Adjustments can be made based on the observed displacement trends and prevailing site conditions.
Structures Inclinometer	Once every 2 weeks	Once daily	Immediately post-construction: Weekly Subsequently: Monthly	

Note: The measurement frequency can be adjusted according to the displacement pattern and site conditions.

Instrumentation is a vital aspect of ensuring the stability and safety of structures. Specific tools, like displacement targets and structural inclinometers, are deployed at precise locations to gauge changes or potential issues. For the most accurate readings, displacement targets need to be strategically positioned. Four points to the left and right of any collapse site are deemed necessary to offer a comprehensive view of the wall’s status. Additionally, structural inclinometers serve as pivotal tools to determine any tilt or shift in apartment walls. For this reason, at least one or two of these instruments should be affixed to each apartment wall, providing consistent monitoring and immediate data in the event of any structural changes.

Table 5 offers a concise summary of the location and quantity for the installation of two primary measurement tools: the Displacement Target and the Structures Inclinometer. Displacement Targets are to be installed on reinforced earth retaining wall sections, specifically at the upper, middle, and lower locations. Instrument placement is determined using both design code recommendations and empirical observations from past case studies, ensuring adequate spatial coverage to capture localized deformations, especially in areas prone to collapse or differential settlement. Across eight designated locations, there will be a total of 24 points, with each location hosting three instruments. This ensures a broad and thorough coverage for monitoring. On the other hand, the Structures Inclinometers are affixed directly to apartment walls. There will be 12 installations in total, distributed across six buildings. Each building will house two inclinometers, providing consistent data on the structural integrity of the apartments. The table serves as a quick reference guide, ensuring that the correct number of instruments are installed at the designated locations.

**Table 5:** Location and Quantity.

Measurement Tool	Installation Location	Total Installation Points	Notes
Displacement Target	On reinforced earth retaining wall sections (at upper, middle, and lower locations)	24 Points across 8 locations	Each location will host 3 instruments, totaling 24 units.
Structures Inclinometer	Affixed to apartment walls	12 installations across 6 buildings	Each building will house 2 inclinometers, summing up to 12 units in total.

Monitoring data from displacement targets and structural inclinometers will serve two critical purposes. Immediately, any readings exceeding predefined threshold values—established from design criteria, historical performance data, or regulatory standards—will trigger site inspections and rapid intervention measures, such as temporary shoring, targeted reinforcement, or load redistribution, to prevent progression to structural failure. In the long term, trend analysis of collected data will guide preventive maintenance planning by identifying zones with progressive deformation, informing the scheduling of localized repairs, grouting, or drainage improvements. This continuous feedback loop ensures that both acute risks and gradual deterioration are systematically managed, extending the service life of the retaining walls and adjacent structures while optimizing maintenance resources.

## Conclusion

The structural integrity and stability of reinforced earth retaining walls are central to urban safety, with failures often causing severe damage to surrounding infrastructure. This study’s in-depth investigation into the collapse site revealed critical causal mechanisms, providing both practical guidance and theoretical implications for geotechnical engineering practice.

This study contributes to the limited body of research on reinforced earth retaining wall failures by integrating field-based DCP testing with MSEW numerical analysis to diagnose the mechanisms of collapse. Unlike previous studies that focus primarily on design or post-failure visual inspection [52–54], our approach quantitatively correlates in-situ strength profiles with stability modeling parameters to capture both the spatial variability of soil conditions and their influence on structural performance. The research also proposes a targeted post-failure measurement and monitoring framework, offering practical guidance for future forensic geotechnical investigations in urban infrastructure settings.

Our field investigation, supported by DCP testing, identified significant heterogeneity in subgrade strength, with notably reduced cone penetration resistance near manhole structures. Such localized weaknesses, exacerbated by pipe breakages and water infiltration, align with previous studies indicating that service utilities can create preferential seepage paths that accelerate structural deterioration [55]. The substantial role of hydrostatic pressure behind the retaining wall—evident from photographic evidence—reinforces the importance of integrating hydrological assessments into geotechnical stability analyses, a factor similarly emphasized in failure analyses by previous works [9,56,57].

Analysis using the MSEW program confirmed that reduced internal friction angles in weakened soil zones critically lowered stability margins. Compared to findings by previous studies [58–60], which demonstrated the effectiveness of soil nailing in enhancing marginal stability, our results highlight the need for combined soil nailing and block façade reinforcement, particularly where DCP values indicate insufficient bearing capacity. For sites with severe local instability, anchored systems or partial wall reconstruction may be warranted, echoing pile optimization and reinforcement strategies discussed by Benmebarek, Benmebarek [61].

The key takeaways for practitioners emphasize that even small-scale heterogeneities can significantly reduce retaining wall stability, making high-resolution, site-specific testing essential; that effective drainage design and waterproofing around utility penetrations are vital for long-term durability; that rehabilitation techniques should be selected based on quantified subgrade conditions, with hybrid reinforcement systems employed where necessary; and that installing displacement targets and structural inclinometers at critical points enables early detection of instability, thereby supporting proactive maintenance.

From a broader research perspective, our results affirm that collapse mechanisms in reinforced earth walls are often a product of interacting factors—geotechnical, structural, and hydrological—rather than a single cause. To advance this field, future work should include large-scale 3D numerical simulations to capture soil–structure–water interactions more accurately, building on methods [62]. Additional in-situ testing, including borehole shear and piezometric monitoring, would help refine stability models and rehabilitation designs.

In conclusion, this study demonstrates that retaining wall failures cannot be effectively prevented through generic design or maintenance protocols. Instead, practitioners must adopt a multi-parameter, site-specific approach that integrates precise subgrade characterization, robust hydrological controls, and adaptive reinforcement strategies, underpinned by continuous monitoring. By situating our findings within the context of contemporary literature and real-world constraints, this research provides a pragmatic framework for both preventing and mitigating future retaining wall failures.

### Limitations and future research directions

This study has certain limitations that should be acknowledged. First, the depth and spatial extent of the DCP tests were constrained by site accessibility and safety considerations. As a result, measurements were limited to accessible wall and ground sections, which may not fully capture subsurface variability in areas beyond the tested zones. Furthermore, the DCP tests, while effective for rapid in-situ strength assessment, provide indirect estimations of soil parameters and may not reflect localized heterogeneities with high precision. Future research could benefit from integrating deeper penetration testing and more spatially extensive surveys to capture broader geotechnical variability. Additionally, complementary experimental methods such as plate load tests or field vane shear tests could enhance the accuracy of soil strength characterization. On the numerical side, advanced simulations incorporating three-dimensional slope stability models, such as those presented by Benmebarek, Benmebarek [61] for pile optimization in slope stabilization, could be adapted to analyze retaining wall behavior under varying load and deformation conditions. Similarly, approaches like those described by Salari, Lezgy-Nazargah [62], which account for rotational and horizontal displacements in foundation systems, could be used to evaluate retaining wall-soil interaction more realistically. By combining enhanced field investigations with advanced numerical modeling, future studies can overcome current limitations, provide more robust design recommendations, and better align practical construction practices with actual geotechnical behavior. Moreover, another limitation of this study is the absence of statistical analysis and sensitivity evaluations to quantitatively assess the robustness of the MSEW and DCP findings. Future research should incorporate probabilistic modeling and parameter sensitivity analyses to strengthen the reliability and generalizability of the results. Finally, this study is based on a single retaining wall failure case, which constrains the generalizability of its findings to other contexts or wall configurations. Future research should incorporate multiple case studies and comparative analyses across varied site conditions to validate the observed mechanisms and strengthen the broader applicability of the conclusions.

## Supporting information

S1 Data. (ZIP)

S2 Retaining Wall 3rd Appendix. (DOCX)
