# Supplementary material for: Field DCP testing, MSEW analysis, and monitoring-based investigation of a reinforced earth retaining wall collapse
Source: PLoS One. 2025 Sep 22;20(9):e0332879. doi: 10.1371/journal.pone.0332879 (PMC12453176; doi:10.1371/journal.pone.0332879)
Supplement: S1 — (ZIP) [file pone.0332879.s001.zip › S2_Data_MSWE Analysis.pdf]

AASHTO 98 ASD DESIGN METHOD
대형 M-블록 H=14.50m 구조 검토

MSEW(3.0): Update # 14.92

PROJECT IDENTIFICATION

Title: 대형 M-블록 H=14.50m 구조 검토
Project Number: [redacted]
Client:
Designer:
Station Number:

Description:

[redacted]

Company's information:

Name: [redacted]
Street:

Telephone #: [redacted]
Fax #:
E-Mail:

Original file path and name: [redacted]

Original date and time of creating this file: Mon Jul 14 23:02:14 2014

PROGRAM MODE: ANALYSIS
of a SIMPLE STRUCTURE
using GEOGRID as reinforcing material.

Copyright ?1998-2013 ADAMA Engineering, Inc.
License number MSEW-302308

SOIL DATA

REINFORCED SOIL

Unit weight, γ	19.0 kN/m ?
Design value of internal angle of friction, ϕ	30.0 ?

RETAINED SOIL

Unit weight, γ	19.0 kN/m ?
Design value of internal angle of friction, ϕ	30.0 ?

FOUNDATION SOIL (Considered as an equivalent uniform soil)

Equivalent unit weight, $\gamma_{equiv.}$	19.0 kN/m ?
Equivalent internal angle of friction, $\phi_{equiv.}$	30.0 ?
Equivalent cohesion, $c_{equiv.}$	0.0 kPa

Water table does not affect bearing capacity

LATERAL EARTH PRESSURE COEFFICIENTS

Ka (internal stability) = 0.2818 (eq. 17 is utilized to calculate Ka for all batters)
(For internal stability user specified $\delta = 29.50°$)
Inclination of internal slip plane, $\psi = 53.79$? (see Fig. 28 in DEMO 82; iψ for all batters).
Ka (external stability) = 0.3333 (if batter is less than 10? Ka is calculated from eq. 16. Otherwise, eq. 17 is utilized)

BEARING CAPACITY

Bearing capacity coefficients (calculated by MSEW): Nc = 30.14 N γ = 22.40

SEISMICITY

Maximum ground acceleration coefficient, A = 0.154
Design acceleration coefficient in Internal Stability: Kh = Am = 0.200
Design acceleration coefficient in External Stability: Kh_d = 0.200 => Kh = Am = 0.200

Kae (Kh > 0) = 0.4596 Kae (Kh = 0) = 0.3201 Δ Kae = 0.1395
Seismic soil-geogrid friction coefficient, F* is 80.0% of its specified static value.

INPUT DATA: Geogrids
(Analysis)

D A T A	Geogrid type #1	Geogrid type #2	Geogrid type #3	Geogrid type #4	Geogrid type #5
Tult [kN/m]	58.8	78.4	98.0	147.0	
Durability reduction factor, RFd	1.08	1.08	1.08	1.08	
Installation-damage reduction factor, RFid	1.10	1.10	1.10	1.10	
Creep reduction factor, RFc	1.20	1.20	1.20	1.20	N/A
Fs-overall for strength	N/A	N/A	N/A	N/A	
Coverage ratio, Rc	1.000	1.000	1.000	1.000	
Friction angle along geogrid-soil interface, ρ	29.50	29.50	29.50	29.50	
Pullout resistance factor, F*	0.90 텔sφ	0.90 텔an	0.90 텔an	0.90 텔an	N/A
Scale-effect correction factor, α	0.8	0.8	0.8	0.8	

Variation of Lateral Earth Pressure Coefficient With Depth

Z	K / Ka
0 m	1.00
1 m	1.00
2 m	1.00
3 m	1.00
4 m	1.00
5 m	1.00
6 m	1.00

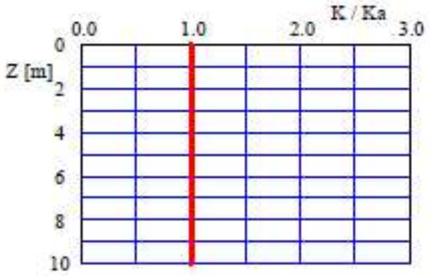

INPUT DATA: Geometry and Surcharge loads (of a SIMPLE STRUCTURE)

Design height, Hd	14.50	[m]	{ Embedded depth is E = 1.50 m, and height above top of finished bottom grade is H = 13.00 m }
Batter, ω	2.0	[deg]	
Backslope, β	0.0	[deg]	
Backslope rise	0.0	[m]	Broken back equivalent angle, I = 0.00? (see Fig. 25 in DEMO 82)

UNIFORM SURCHARGE

Uniformly distributed dead load is 13.0 [kPa]

ANALYZED REINFORCEMENT LAYOUT:

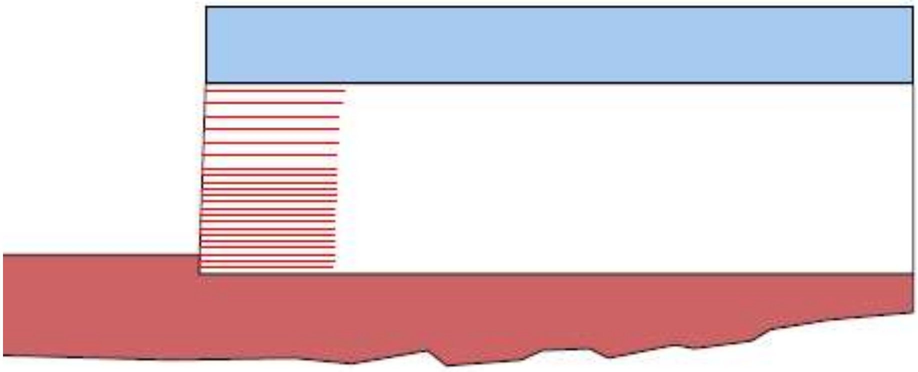

SCALE:

0 2 4 6 8 10 [m]

Copyright ?1998-2013 ADAMA Engineering, Inc.
License number MSEW-302308

ANALYSIS: CALCULATED FACTORS (Static conditio Bearing capacity, Fs = 4.63, Meyerhof stress = 362.87 kPa.
Foundation Interface: Direct sliding, Fs = 2.271, Eccentricity, e/L = 0.1128, Fs-overturning = 4.08

GEOGRID				CONNECTION							
#	Elevation [m]	Length [m]	Type #	Fs-overall [pullout resistance]	Fs-overall [connection break]	Fs-overall [geogrid strength]	Geogrid strength Fs	Pullout resistance Fs	Direct sliding Fs	Eccentricity e/L	Product name
1	0.50	10.20	4	2.51	1.48	1.95	1.954	43.389	2.304	0.1049	15T
2	1.00	10.20	4	3.93	2.32	3.06	3.061	63.480	2.388	0.0973	15T
3	1.50	10.20	4	4.07	2.40	3.17	3.173	61.149	2.477	0.0901	15T
4	2.00	10.20	4	4.22	2.49	3.29	3.293	58.818	2.574	0.0830	15T
5	2.50	10.20	4	4.39	2.59	3.42	3.423	56.487	2.678	0.0763	15T
6	3.00	10.20	4	4.57	2.70	3.56	3.563	54.156	2.792	0.0699	15T
7	3.50	10.20	4	4.77	2.81	3.72	3.716	51.825	2.914	0.0637	15T
8	4.00	10.20	4	4.98	2.94	3.88	3.882	49.494	3.048	0.0579	15T
9	4.50	10.20	4	5.21	3.08	4.06	4.064	47.163	3.195	0.0523	15T
10	5.00	10.20	3	3.65	2.15	2.84	2.842	44.832	3.356	0.0470	10T
11	5.50	10.20	3	3.83	2.26	2.99	2.989	42.501	3.534	0.0420	10T
12	6.00	10.20	3	4.04	2.39	3.15	3.152	40.170	3.732	0.0373	10T
13	6.50	10.20	3	4.28	2.52	3.33	3.333	37.870	3.953	0.0328	10T
14	7.00	10.20	3	4.54	2.68	3.54	3.537	35.539	4.201	0.0286	10T
15	7.50	10.20	3	4.83	2.85	3.77	3.767	33.208	4.483	0.0247	10T
16	8.00	10.20	3	3.51	2.07	2.73	2.733	20.949	4.804	0.0211	10T
17	9.00	10.20	3	3.00	1.77	2.34	2.340	13.108	5.609	0.0147	10T
18	10.00	10.20	2	2.87	1.69	2.23	2.233	10.777	6.738	0.0094	8T
19	11.00	10.20	2	3.55	2.10	2.77	2.767	8.446	8.443	0.0053	8T
20	12.00	10.20	1	3.50	2.07	2.73	2.727	6.115	11.341	0.0022	6T
21	13.00	10.50	1	5.10	3.01	3.98	3.975	4.798	18.035	0.0002	6T
22	14.00	10.50	1	7.53	5.55	7.33	7.333	2.467	45.123	-0.0004	6T

ANALYSIS: CALCULATED FACTORS (Seismic conditi Bearing capacity, Fs = 1.95, Meyerhof stress = 558.70 kPa.
Foundation Interface: Direct sliding, Fs = 1.321, Eccentricity, e/L = 0.2485, Fs-overturning = 1.96

GEOGRID				CONNECTION							
#	Elevation [m]	Length [m]	Type #	Fs-overall [pullout resistance]	Fs-overall [connection break]	Fs-overall [geogrid strength]	Geogrid strength Fs	Pullout resistance Fs	Direct sliding Fs	Eccentricity e/L	Product name
1	0.50	10.20	4	1.43	1.17	1.47	1.465	24.780	1.342	0.2311	15T
2	1.00	10.20	4	1.96	1.66	2.03	2.035	31.633	1.392	0.2144	15T
3	1.50	10.20	4	2.03	1.73	2.11	2.110	30.489	1.447	0.1983	15T
4	2.00	10.20	4	2.11	1.79	2.19	2.191	29.344	1.506	0.1828	15T
5	2.50	10.20	4	2.19	1.86	2.28	2.279	28.200	1.570	0.1680	15T
6	3.00	10.20	4	2.28	1.94	2.37	2.374	27.055	1.639	0.1538	15T
7	3.50	10.20	4	2.38	2.03	2.48	2.477	25.910	1.714	0.1403	15T
8	4.00	10.20	4	2.49	2.12	2.59	2.590	24.766	1.797	0.1274	15T
9	4.50	10.20	4	2.61	2.22	2.71	2.713	23.621	1.888	0.1151	15T
10	5.00	10.20	3	1.83	1.55	1.90	1.899	22.476	1.988	0.1034	10T
11	5.50	10.20	3	1.92	1.63	2.00	1.999	21.331	2.100	0.0924	10T
12	6.00	10.20	3	2.03	1.72	2.11	2.110	20.186	2.224	0.0820	10T
13	6.50	10.20	3	2.15	1.83	2.23	2.235	19.056	2.364	0.0722	10T
14	7.00	10.20	3	2.29	1.94	2.37	2.374	17.911	2.522	0.0630	10T
15	7.50	10.20	3	2.44	2.07	2.53	2.533	16.765	2.703	0.0545	10T
16	8.00	10.20	3	2.01	1.65	2.06	2.057	12.018	2.911	0.0466	10T
17	9.00	10.20	3	1.87	1.49	1.89	1.889	8.149	3.440	0.0326	10T
18	10.00	10.20	2	1.79	1.43	1.81	1.809	6.728	4.202	0.0211	8T
19	11.00	10.20	2	2.23	1.78	2.25	2.254	5.306	5.392	0.0121	8T
20	12.00	10.20	1	2.22	1.77	2.24	2.241	3.882	7.517	0.0054	6T
21	13.00	10.50	1	3.15	2.52	3.19	3.186	2.959	12.776	0.0012	6T
22	14.00	10.50	1	4.70	4.69	5.94	5.936	1.539	37.272	-0.0003	6T

GLOBAL/COMPOUND STABILITY ANALYSIS (Using Demo 82 method and ROR = 1.0)

STATIC CONDITIONS: For the specified search grid, the calculated minimum Fs is 1.787
(it corresponds to a critical circle at Xc = -1.45, Yc = 21.75 and R = 25.06 [m]).

SEISMIC CONDITIONS: For the specified search grid, the calculated minimum Fs is 1.330
(it corresponds to a critical circle at Xc = -1.45, Yc = 27.55 and R = 30.23 [m]).

Copyright ?1998-2013 ADAMA Engineering, Inc.
License number MSEW-302308

BEARING CAPACITY for GIVEN LAYOUT

	STATIC	SEISMIC	UNITS
(Water table does not affect bearing capacity)			
Ultimate bearing capacity, q_{ult}	1681.1	1091.9	[kPa]
Meyerhof stress, σ_v	362.87	558.7	[kPa]
Eccentricity, e	1.15	2.53	[m]
Eccentricity, e/L	0.113	0.249	
Fs calculated	4.63	1.95	
Base length	10.20	10.20	[m]

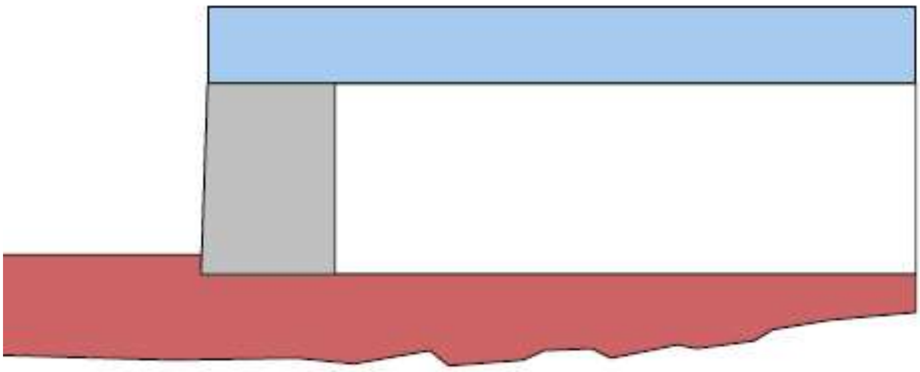

SCALE:

0 2 4 6 8 10 [m]

Copyright ?1998-2013 ADAMA Engineering, Inc.
License number MSEW-302308

DIRECT SLIDING for GIVEN LAYOUT (for GEOGRID reinforcements)

Along reinforced and foundation soils interface: Fs-static = 2.271 and Fs-seismic = 1.321

#	Geogrid Elevation [m]	Geogrid Length [m]	Fs Static	Fs Seismic	Geogrid Type #	Product name
1	0.50	10.20	2.304	1.342	4	15T
2	1.00	10.20	2.388	1.392	4	15T
3	1.50	10.20	2.477	1.447	4	15T
4	2.00	10.20	2.574	1.506	4	15T
5	2.50	10.20	2.678	1.570	4	15T
6	3.00	10.20	2.792	1.639	4	15T
7	3.50	10.20	2.914	1.714	4	15T
8	4.00	10.20	3.048	1.797	4	15T
9	4.50	10.20	3.195	1.888	4	15T
10	5.00	10.20	3.356	1.988	3	10T
11	5.50	10.20	3.534	2.100	3	10T
12	6.00	10.20	3.732	2.224	3	10T
13	6.50	10.20	3.953	2.364	3	10T
14	7.00	10.20	4.201	2.522	3	10T
15	7.50	10.20	4.483	2.703	3	10T
16	8.00	10.20	4.804	2.911	3	10T
17	9.00	10.20	5.609	3.440	3	10T
18	10.00	10.20	6.738	4.202	2	8T
19	11.00	10.20	8.443	5.392	2	8T
20	12.00	10.20	11.341	7.517	1	6T
21	13.00	10.50	18.035	12.776	1	6T
22	14.00	10.50	45.123	37.272	1	6T

ECCENTRICITY for GIVEN LAYOUT

At interface with foundation: e/L static = 0.1128, e/L seismic = 0.2485; Overturning: Fs-static = 4.08, Fs-seismic = 1.96

#	Geogrid Elevation [m]	Geogrid Length [m]	e / L Static	e / L Seismic	Geogrid Type #	Product name
1	0.50	10.20	0.1049	0.2311	4	15T
2	1.00	10.20	0.0973	0.2144	4	15T
3	1.50	10.20	0.0901	0.1983	4	15T
4	2.00	10.20	0.0830	0.1828	4	15T
5	2.50	10.20	0.0763	0.1680	4	15T
6	3.00	10.20	0.0699	0.1538	4	15T
7	3.50	10.20	0.0637	0.1403	4	15T
8	4.00	10.20	0.0579	0.1274	4	15T
9	4.50	10.20	0.0523	0.1151	4	15T
10	5.00	10.20	0.0470	0.1034	3	10T
11	5.50	10.20	0.0420	0.0924	3	10T
12	6.00	10.20	0.0373	0.0820	3	10T
13	6.50	10.20	0.0328	0.0722	3	10T
14	7.00	10.20	0.0286	0.0630	3	10T
15	7.50	10.20	0.0247	0.0545	3	10T
16	8.00	10.20	0.0211	0.0466	3	10T
17	9.00	10.20	0.0147	0.0326	3	10T
18	10.00	10.20	0.0094	0.0211	2	8T
19	11.00	10.20	0.0053	0.0121	2	8T
20	12.00	10.20	0.0022	0.0054	1	6T
21	13.00	10.50	0.0002	0.0012	1	6T
22	14.00	10.50	-0.0004	-0.0003	1	6T

Copyright ?1998-2013 ADAMA Engineering, Inc. License number MSEW-302308

RESULTS for STRENGTH

Live Load included in calculating Tmax

#	Geogrid Elevation [m]	Tavailable [kN/m]	Tmax [kN/m]	Tmd [kN/m]	Specified minimum Fs-overall static	Actual calculated Fs-overall static	Specified minimum Fs-overall seismic	Actual calculated Fs-overall seismic	Product name
1	0.50	103.1	52.76	21.14	N/A	1.954	N/A	1.465	15T
2	1.00	103.1	33.69	20.39	N/A	3.061	N/A	2.035	15T
3	1.50	103.1	32.50	19.65	N/A	3.173	N/A	2.110	15T
4	2.00	103.1	31.31	18.90	N/A	3.293	N/A	2.191	15T
5	2.50	103.1	30.13	18.15	N/A	3.423	N/A	2.279	15T
6	3.00	103.1	28.94	17.40	N/A	3.563	N/A	2.374	15T
7	3.50	103.1	27.75	16.65	N/A	3.716	N/A	2.477	15T
8	4.00	103.1	26.56	15.91	N/A	3.882	N/A	2.590	15T
9	4.50	103.1	25.38	15.16	N/A	4.064	N/A	2.713	15T
10	5.00	68.7	24.19	14.41	N/A	2.842	N/A	1.899	10T
11	5.50	68.7	23.00	13.66	N/A	2.989	N/A	1.999	10T
12	6.00	68.7	21.81	12.91	N/A	3.152	N/A	2.110	10T
13	6.50	68.7	20.63	12.17	N/A	3.333	N/A	2.235	10T
14	7.00	68.7	19.44	11.42	N/A	3.537	N/A	2.374	10T
15	7.50	68.7	18.25	10.67	N/A	3.767	N/A	2.533	10T
16	8.00	68.7	25.15	9.92	N/A	2.733	N/A	2.057	10T
17	9.00	68.7	29.38	8.43	N/A	2.340	N/A	1.889	10T
18	10.00	55.0	24.63	6.93	N/A	2.233	N/A	1.809	8T
19	11.00	55.0	19.88	5.43	N/A	2.767	N/A	2.254	8T
20	12.00	41.2	15.13	3.94	N/A	2.727	N/A	2.241	6T
21	13.00	41.2	10.38	3.08	N/A	3.975	N/A	3.186	6T
22	14.00	41.2	5.63	1.59	N/A	7.333	N/A	5.936	6T

RESULTS for PULLOUT

Live Load included in calculating Tmax

#	Geogrid Elevation [m]	Coverage Ratio	Tmax [kN/m]	Tmd [kN/m]	Le [m]	La [m]	Avail.Static Pullout, Pr [kN/m]	Specified Static Fs	Actual Static Fs	Avail.Seism. Pullout, Pr [kN/m]	Specified Seismic Fs	Actual Seismic Fs
1	0.50	1.000	52.76	21.14	9.85	0.35	2289.1	N/A	43.389	1831.3	N/A	24.780
2	1.00	1.000	33.69	20.39	9.50	0.70	2138.5	N/A	63.480	1710.8	N/A	31.633
3	1.50	1.000	32.50	19.65	9.15	1.05	1987.3	N/A	61.149	1589.9	N/A	30.489
4	2.00	1.000	31.31	18.90	8.81	1.39	1841.7	N/A	58.818	1473.4	N/A	29.344
5	2.50	1.000	30.13	18.15	8.46	1.74	1701.7	N/A	56.487	1361.3	N/A	28.200
6	3.00	1.000	28.94	17.40	8.11	2.09	1567.1	N/A	54.156	1253.7	N/A	27.055
7	3.50	1.000	27.75	16.65	7.76	2.44	1438.1	N/A	51.825	1150.5	N/A	25.910
8	4.00	1.000	26.56	15.91	7.41	2.79	1314.7	N/A	49.494	1051.8	N/A	24.766
9	4.50	1.000	25.38	15.16	7.06	3.14	1196.8	N/A	47.163	957.4	N/A	23.621
10	5.00	1.000	24.19	14.41	6.71	3.49	1084.4	N/A	44.832	867.5	N/A	22.476
11	5.50	1.000	23.00	13.66	6.37	3.83	977.5	N/A	42.501	782.0	N/A	21.331
12	6.00	1.000	21.81	12.91	6.02	4.18	876.2	N/A	40.170	701.0	N/A	20.186
13	6.50	1.000	20.63	12.17	5.67	4.53	781.1	N/A	37.870	624.9	N/A	19.056
14	7.00	1.000	19.44	11.42	5.32	4.88	690.8	N/A	35.539	552.6	N/A	17.911
15	7.50	1.000	18.25	10.67	4.97	5.23	606.0	N/A	33.208	484.8	N/A	16.765
16	8.00	1.000	25.15	9.92	4.62	5.58	526.8	N/A	20.949	421.5	N/A	12.018
17	9.00	1.000	29.38	8.43	3.93	6.27	385.0	N/A	13.108	308.0	N/A	8.149
18	10.00	1.000	24.63	6.93	3.23	6.97	265.4	N/A	10.777	212.3	N/A	6.728
19	11.00	1.000	19.88	5.43	2.53	7.67	167.9	N/A	8.446	134.3	N/A	5.306
20	12.00	1.000	15.13	3.94	1.83	8.37	92.5	N/A	6.115	74.0	N/A	3.882
21	13.00	1.000	10.38	3.08	1.44	9.06	49.8	N/A	4.798	39.8	N/A	2.959
22	14.00	1.000	5.63	1.59	0.74	9.76	13.9	N/A	2.467	11.1	N/A	1.539

Copyright ?1998-2013 ADAMA Engineering, Inc. License number MSEW-302308

RESULTS for CONNECTION (static conditions)
Live Load included in calculating Tmax

#	Geogrid Elevation [m]	Connection force, To [kN/m]	Reduction factor for connection break, CRu	Reduction factor for connection pullout, CRs	Available connection strength, Tc-break criterion [kN/m]	Available connection strength, Tc-pullout criterion [kN/m]	Available Geogrid strength, Tavailable [kN/m]	Fs-overall connection break Specified	Fs-overall connection break Actual	Fs-overall connection pullout Specified	Fs-overall connection pullout Actual	Fs-overall Geogrid strength Specified	Fs-overall Geogrid strength Actual	Product name
1	0.50	52.8	0.90	0.90	78.1	132.3	103.1	N/A	1.48	N/A	2.51	N/A	1.95	15T
2	1.00	33.7	0.90	0.90	78.1	132.3	103.1	N/A	2.32	N/A	3.93	N/A	3.06	15T
3	1.50	32.5	0.90	0.90	78.1	132.3	103.1	N/A	2.40	N/A	4.07	N/A	3.17	15T
4	2.00	31.3	0.90	0.90	78.1	132.3	103.1	N/A	2.49	N/A	4.22	N/A	3.29	15T
5	2.50	30.1	0.90	0.90	78.1	132.3	103.1	N/A	2.59	N/A	4.39	N/A	3.42	15T
6	3.00	28.9	0.90	0.90	78.1	132.3	103.1	N/A	2.70	N/A	4.57	N/A	3.56	15T
7	3.50	27.8	0.90	0.90	78.1	132.3	103.1	N/A	2.81	N/A	4.77	N/A	3.72	15T
8	4.00	26.6	0.90	0.90	78.1	132.3	103.1	N/A	2.94	N/A	4.98	N/A	3.88	15T
9	4.50	25.4	0.90	0.90	78.1	132.3	103.1	N/A	3.08	N/A	5.21	N/A	4.06	15T
10	5.00	24.2	0.90	0.90	52.1	88.2	68.7	N/A	2.15	N/A	3.65	N/A	2.84	10T
11	5.50	23.0	0.90	0.90	52.1	88.2	68.7	N/A	2.26	N/A	3.83	N/A	2.99	10T
12	6.00	21.8	0.90	0.90	52.1	88.2	68.7	N/A	2.39	N/A	4.04	N/A	3.15	10T
13	6.50	20.6	0.90	0.90	52.1	88.2	68.7	N/A	2.52	N/A	4.28	N/A	3.33	10T
14	7.00	19.4	0.90	0.90	52.1	88.2	68.7	N/A	2.68	N/A	4.54	N/A	3.54	10T
15	7.50	18.3	0.90	0.90	52.1	88.2	68.7	N/A	2.85	N/A	4.83	N/A	3.77	10T
16	8.00	25.1	0.90	0.90	52.1	88.2	68.7	N/A	2.07	N/A	3.51	N/A	2.73	10T
17	9.00	29.4	0.90	0.90	52.1	88.2	68.7	N/A	1.77	N/A	3.00	N/A	2.34	10T
18	10.00	24.6	0.90	0.90	41.7	70.6	55.0	N/A	1.69	N/A	2.87	N/A	2.23	8T
19	11.00	19.9	0.90	0.90	41.7	70.6	55.0	N/A	2.10	N/A	3.55	N/A	2.77	8T
20	12.00	15.1	0.90	0.90	31.2	52.9	41.2	N/A	2.07	N/A	3.50	N/A	2.73	6T
21	13.00	10.4	0.90	0.90	31.2	52.9	41.2	N/A	3.01	N/A	5.10	N/A	3.98	6T
22	14.00	5.6	0.90	0.72	31.2	42.3	41.2	N/A	5.55	N/A	7.53	N/A	7.33	6T

RESULTS for CONNECTION (seismic conditions)
Live Load included in calculating Tmax

#	Geogrid Elevation [m]	Connection force, To [kN/m]	Reduction factor for connection break, CRu	Reduction factor for connection pullout, CRs	Available connection strength, Tc-break criterion [kN/m]	Available connection strength, Tc-pullout criterion [kN/m]	Available Geogrid strength, Tavailable [kN/m]	Fs-overall connection break Specified	Fs-overall connection break Actual	Fs-overall connection pullout Specified	Fs-overall connection pullout Actual	Fs-overall Geogrid strength Specified	Fs-overall Geogrid strength Actual	Product name
1	0.50	73.9	0.90	0.72	78.1	105.8	103.1	N/A	1.17	N/A	1.43	N/A	1.47	15T
2	1.00	54.1	0.90	0.72	78.1	105.8	103.1	N/A	1.66	N/A	1.96	N/A	2.03	15T
3	1.50	52.1	0.90	0.72	78.1	105.8	103.1	N/A	1.73	N/A	2.03	N/A	2.11	15T
4	2.00	50.2	0.90	0.72	78.1	105.8	103.1	N/A	1.79	N/A	2.11	N/A	2.19	15T
5	2.50	48.3	0.90	0.72	78.1	105.8	103.1	N/A	1.86	N/A	2.19	N/A	2.28	15T
6	3.00	46.3	0.90	0.72	78.1	105.8	103.1	N/A	1.94	N/A	2.28	N/A	2.37	15T
7	3.50	44.4	0.90	0.72	78.1	105.8	103.1	N/A	2.03	N/A	2.38	N/A	2.48	15T
8	4.00	42.5	0.90	0.72	78.1	105.8	103.1	N/A	2.12	N/A	2.49	N/A	2.59	15T
9	4.50	40.5	0.90	0.72	78.1	105.8	103.1	N/A	2.22	N/A	2.61	N/A	2.71	15T
10	5.00	38.6	0.90	0.72	52.1	70.6	68.7	N/A	1.55	N/A	1.83	N/A	1.90	10T
11	5.50	36.7	0.90	0.72	52.1	70.6	68.7	N/A	1.63	N/A	1.92	N/A	2.00	10T
12	6.00	34.7	0.90	0.72	52.1	70.6	68.7	N/A	1.72	N/A	2.03	N/A	2.11	10T
13	6.50	32.8	0.90	0.72	52.1	70.6	68.7	N/A	1.83	N/A	2.15	N/A	2.23	10T
14	7.00	30.9	0.90	0.72	52.1	70.6	68.7	N/A	1.94	N/A	2.29	N/A	2.37	10T
15	7.50	28.9	0.90	0.72	52.1	70.6	68.7	N/A	2.07	N/A	2.44	N/A	2.53	10T
16	8.00	35.1	0.90	0.72	52.1	70.6	68.7	N/A	1.65	N/A	2.01	N/A	2.06	10T
17	9.00	37.8	0.90	0.72	52.1	70.6	68.7	N/A	1.49	N/A	1.87	N/A	1.89	10T
18	10.00	31.6	0.90	0.72	41.7	56.4	55.0	N/A	1.43	N/A	1.79	N/A	1.81	8T
19	11.00	25.3	0.90	0.72	41.7	56.4	55.0	N/A	1.78	N/A	2.23	N/A	2.25	8T
20	12.00	19.1	0.90	0.72	31.2	42.3	41.2	N/A	1.77	N/A	2.22	N/A	2.24	6T
21	13.00	13.5	0.90	0.72	31.2	42.3	41.2	N/A	2.52	N/A	3.15	N/A	3.19	6T
22	14.00	7.2	0.90	0.58	31.2	33.9	41.2	N/A	4.69	N/A	4.70	N/A	5.94	6T

Copyright ?1998-2013 ADAMA Engineering, Inc. License number MSEW-302308

GLOBAL/COMPOUND STABILITY ANALYSIS (Using Demo 82 method and ROR = 1.0)

A horizontal seismic coefficient, Kh='A', equal to 0.154 has been applied.
The seismic force is applied at the center of the sliding mass.

STATIC CONDITIONS:
For the specified search grid, the calculated minimum Fs is 1.787
(it corresponds to a critical circle at Xc = -1.45, Yc = 21.75 and R = 25.06 [m] where (x=0, y=0) is taken at the TOE or Xc = 18.55, Yc = 121.75 and R = 25.06 [m] when the terrain coordinate system is used as shown in the table below.)

SEISMIC CONDITIONS:
For the specified search grid, the calculated minimum Fs is 1.330
(it corresponds to a critical circle at Xc = -1.45, Yc = 27.55 and R = 30.23 [m] where (x=0, y=0) is taken at the TOE or Xc = 18.55, Yc = 127.55 and R = 30.23 [m] when the terrain coordinate system is used as shown in the table below.)

TERRAIN/WATER PROFILE

Point	#1	#2	#3	#4	#5	#6	#7	#8	#9	#10	#11
Soil layer #1:	γ = 19.00 [kN/m ?			ϕ = 30.0?		c = 0.00 [kN/m ?					
x [m]	0.0	2.0	4.0	6.0	8.0	84.0	86.0	88.0	90.0	95.0	100.0
y [m]	101.5	101.5	101.5	101.5	101.5	100.0	100.0	100.0	100.0	120.0	120.0
Soil layer #2	γ = 20.00 [kN/m ?			ϕ = 25.0?		c = 0.00 [kN/m ?					
x [m]	0.0	2.0	4.0	6.0	8.0	84.0	86.0	88.0	90.0	95.0	100.0
y [m]	95.0	95.0	95.0	95.0	95.0	95.0	95.0	95.0	95.0	115.0	115.0
Soil layer #3:	γ = 18.90 [kN/m ?			ϕ = 30.0?		c = 0.00 [kN/m ?					
x [m]	0.0	2.0	4.0	6.0	8.0	84.0	86.0	88.0	90.0	95.0	100.0
y [m]	90.0	90.0	90.0	90.0	90.0	90.0	90.0	90.0	90.0	110.0	110.0
Soil layer #4:	γ = 18.90 [kN/m ?			ϕ = 30.0?		c = 0.00 [kN/m ?					
x [m]	0.0	2.0	4.0	6.0	8.0	84.0	86.0	88.0	90.0	95.0	100.0
y [m]	85.0	85.0	85.0	85.0	85.0	85.0	85.0	85.0	85.0	105.0	105.0

Copyright ?1998-2013 ADAMA Engineering, Inc. License number MSEW-302308
