# Supplementary material for: Field DCP testing, MSEW analysis, and monitoring-based investigation of a reinforced earth retaining wall collapse
Source: PLoS One. 2025 Sep 22;20(9):e0332879. doi: 10.1371/journal.pone.0332879 (PMC12453176; doi:10.1371/journal.pone.0332879)
Supplement: S2 — (DOCX) [file pone.0332879.s002.docx]

Appendix A1

The portable DCP test, a critical tool in geotechnical engineering, provides valuable insights into the stratigraphy and strength of the subsurface. The portable DCP tester, indigenously developed, is versatile, enabling configurations akin to its German and Japanese counterparts by judiciously pairing cones and hammers1. A notable innovation in this apparatus is the verticality holding device. Traditional testers often struggle to maintain verticality, especially as depth increases, which can compromise data reliability. This new feature addresses this challenge. Furthermore, for sites with accessibility issues, this device can be effortlessly detached and reattached.
